# Genetic variation in *ZmTIP1* contributes to root hair elongation and drought tolerance in maize

**DOI:** 10.1111/pbi.13290

**Published:** 2019-11-19

**Authors:** Xiaomin Zhang, Yue Mi, Hude Mao, Shengxue Liu, Limei Chen, Feng Qin

**Affiliations:** ^1^ Key Laboratory of Plant Molecular Physiology Institute of Botany Chinese Academy of Sciences Beijing China; ^2^ University of Chinese Academy of Sciences Beijing China; ^3^ State Key Laboratory of Plant Physiology and Biochemistry College of Biological Sciences China Agricultural University Beijing China; ^4^ State Key Laboratory of Crop Stress Biology for Arid Areas College of Plant Protection Northwest A&F University Shaanxi China; ^5^ Center for Crop Functional Genomics and Molecular Breeding China Agricultural University Beijing China

**Keywords:** maize (*Zea mays*), drought tolerance, *S*‐acylation, root hair elongation, ZmCPK9

## Abstract

Drought is a major abiotic stress that threatens maize production globally. A previous genome‐wide association study identified a significant association between the natural variation of *ZmTIP1* and the drought tolerance of maize seedlings. Here, we report on comprehensive genetic and functional analysis, indicating that *ZmTIP1,* which encodes a functional *S*‐acyltransferase, plays a positive role in regulating the length of root hairs and the level of drought tolerance in maize. We show that enhancing *ZmTIP1* expression in transgenic Arabidopsis and maize increased root hair length, as well as plant tolerance to water deficit. In contrast, *ZmTIP1* transposon‐insertional mutants displayed the opposite phenotype. A calcium‐dependent protein kinase, ZmCPK9, was identified as a substrate protein of ZmTIP1, and ZmTIP1‐mediated palmitoylation of two cysteine residues facilitated the ZmCPK9 PM association. The results of this research enrich our knowledge about ZmTIP1‐mediated protein *S*‐acylation modifications in relation to the regulation of root hair elongation and drought tolerance. Additionally, the identification of a favourable allele of *ZmTIP1* also provides a valuable genetic resource or selection target for the genetic improvement of maize.

## Introduction

Maize is an essential cereal crop that is grown worldwide, providing a major resource for food, feed, and industrial materials. The production of maize, however, is sensitive to environmental stresses, such as drought, that are due to insufficient and/or unpredictable rainfall and global climate change. A considerable number of studies in maize have demonstrated that water scarcity causes a reduction in plant height, early senescence, asynchronous flowering of tassels and ears, and kernel abortion. Severe episodes of drought at the seedling stage are especially detrimental to plant viability and development. Therefore, it is important to identify the genetic components contributing to maize drought tolerance so that the trait can be genetically improved (Nakashima *et al.*, 2014; Xiong *et al.*, 2002). However, due to the complexity of the drought tolerance trait, which is associated with a variety of physiological parameters and controlled by multiple genes, improving drought tolerance in maize has been difficult. As a cross‐pollinated crop, maize is thought to be an ideal model plant for conducting association studies because of its great genetic diversity and genome‐wide rapid linkage disequilibrium (LD) decay (Gore *et al.*, 2009; Yan *et al.*, 2009). Recent progress in genome‐wide association studies (GWAS) has facilitated the genetic dissection of several complex traits, including β‐carotene concentration, oil biosynthesis in maize kernels, photoperiod sensitivity and seedling drought tolerance (Li *et al.*, 2013; Wang *et al.*, 2016; Yan *et al.*, 2010; Yang *et al.*, 2013). Although GWAS provides valuable information about the genetic loci underlying a specific trait, the accurate identification of the underlying allelic variation responsible for a specific trait, as well as the functional mechanism, typically demands additional studies.

Vascular plants utilize root hairs to enhance water uptake. The root hair zone, located between the root apical meristem and maturation zone, is the region that is most permeable to water due to the proliferation of root hairs and the existence of mature, functional water conduits within the root. A comparison of *brb* (*bald root barley*), a barley hairless mutant, with wild‐type (WT) plants using magnetic resonance imaging (MRI) revealed significant depletion of soil water in the root hair zone in WT plants but not in the *brb* mutant (Segal *et al.*, 2008). Transcriptome analysis of *rhl1.a*, another barley root hairless mutant, demonstrated that the expression of stress response genes associated with ABA anabolism, stomatal movement and cell wall synthesis was not activated in response to water stress in the *rhl1.a* mutant as they were in WT plants. Furthermore, processes associated with heat and high‐light intensity stress, as well as the production of hydrogen peroxide, were specifically up‐regulated in leaves of *rhl1.a* plants (Kwasniewski *et al.*, 2016). These data suggest that root hairs may play an important role in sensing and responding to drought stress.

The molecular network involved in root hair formation, from initiation to elongation, has been well studied in Arabidopsis (Balcerowicz *et al.*, 2015; Bruex *et al.*, 2012; Cui *et al.*, 2018; Grierson *et al.*, 2014; Gu and Nielsen, 2013; Salazar‐Henao *et al.*, 2016). In maize, however, six mutants (*roothairless*, *rth1*–*rth6*) that display altered root hair elongation phenotypes have been identified, and four of the responsible genes have been cloned. Notably, *rth1* encodes a SEC3‐like protein that plays a key role in polar exocytosis, mediating the exocytotic tip growth of root hairs (Wen *et al.*, 2005). The gene *rth3* encodes a putative glycosylphosphatidylinositol (GPI)‐anchored, monocot‐specific COBRA‐like protein, and the *rth3* mutant exhibits a significant decrease in grain yield (Hochholdinger *et al.*, 2008). The gene *rth5* encodes a monocot‐specific NADPH oxidase, which is involved in both root hair initiation and elongation as the mutant exhibits reduced density and length of root hairs (Nestler *et al.*, 2014). The gene *rth6* encodes a cellulose synthase‐like D 5 (CslD5) protein, which is responsible for cell wall biosynthesis. Root hairs in the *rth6* mutant exhibited arrested growth after bulge formation and prior to tip growth (Li, *et al.*, 2016a). Recently, *ZmLRL5*, a basic helix–loop–helix (bHLH) transcription factor gene, with the highest expression in root hair among this gene family, was demonstrated to play a role in root hair elongation in maize (Wang *et al.*, 2019).

*S*‐acylation, more commonly known as *S*‐palmitoylation, is a reversible posttranslational modification (PTM) that adds a 16‐carbon palmitate to a specific cysteine residue via a thioester bond. A proteomic analysis revealed that there are up to 600 *S*‐acylated proteins in Arabidopsis root cell cultures that function in plant growth, development and stress responses. The specific PATs responsible for these different functional activities, however, remain largely unknown (Hemsley *et al.*, 2013b). Tip Growth Defective 1 (TIP1)/PAT24 is the first *S*‐acyltransferase to be identified in plants, with the mutation of this gene resulting in an impairment in root hair elongation but not initiation (Hemsley *et al.*, 2005). The target substrate protein of this PAT is still unknown. PAT10 is predominantly localized on Golgi and tonoplast membranes. Mutation of this gene results in pleiotropic defects in plant development, and an alteration in the localization of three calcineurin B‐like proteins (CBL2/3/6) to the tonoplast, probably through protein palmitoylation (Qi *et al.*, 2013; Zhou *et al.*, 2013). PAT4 was also reported to be potentially responsible for ROP2 *S*‐acylation in root hairs (Wan *et al.*, 2017). PAT13 and PAT14 are involved in regulating precocious leaf senescence, though the mechanism is unclear (Lai *et al.*, 2015; Li *et al.*, 2016b). These results imply that protein *S*‐acylation plays an important role in a wide array of biological processes and that identifying the substrate proteins targeted by specific PATs is key to understanding their biological function *in planta*.

In a genome‐wide association study that we previously conducted, a significant association was identified between the genetic variation in *ZmTIP1* and the drought tolerance of maize seedlings. In the current study, we reported the results of a comprehensive genetic and functional characterization of *ZmTIP1*, which revealed that genetic variations in the promoter region but not the protein‐coding region are responsible for the gene functional variation in drought tolerance. Enhancing *ZmTIP1* expression in transgenic Arabidopsis and maize increased root hair length and drought tolerance, while *ZmTIP1* loss of function exhibited the reverse effects. In addition, we identified a calcium‐dependent protein kinase (ZmCPK9) as the substrate of ZmTIP1 for *S*‐acyl modification, which facilitates the association of ZmCPK9 with the plasma membrane.

## Results

### Association analysis of* ZmTIP1* with drought tolerance of maize seedlings

**Figure 1:**
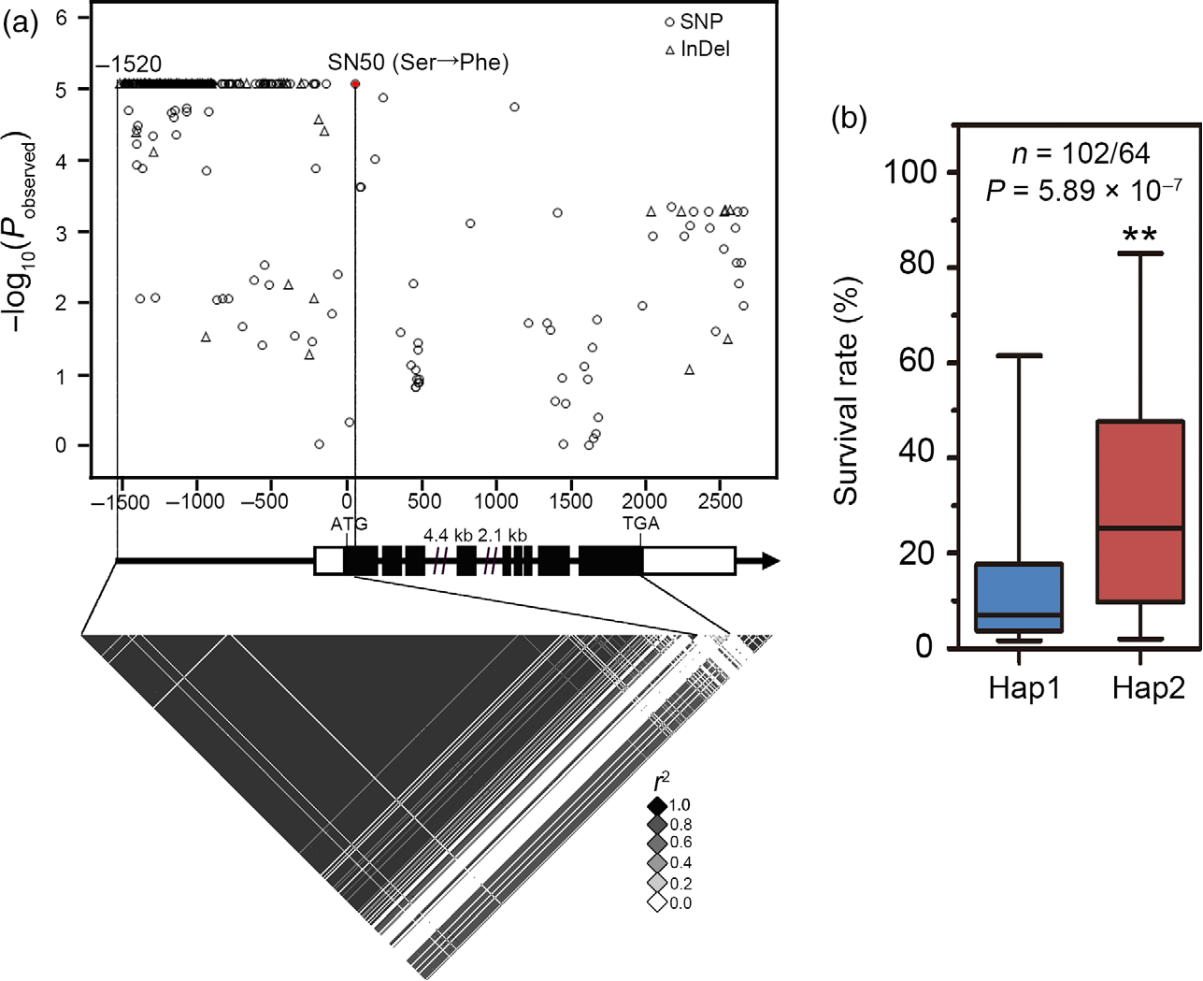
Genetic variation in *ZmTIP1* is associated with drought tolerance in maize seedlings. (a) Association analysis of the genetic variation in *ZmTIP1* with survival rate (SR) of maize seedling subjected to drought stress. Dots denote SNPs, and triangles represent InDels. The *p*‐value is shown on a ‐log10 scale. A non‐synonymous variant (SNP50) is highlighted in red. InDel‐1520 and SNP50 are connected to the pairwise LD diagram with a solid line, highlighting the high number of variants in this region is in complete LD with SNP50 (*r^2^* = 1). Lastly, 5' and 3'‐UTRs and exons of *ZmTIP1* are shown as open and filled boxes. The gene promoter and introns are shown as dark lines. (b) The SR distribution of inbred lines of the two haplotypes is displayed in the box plot. *n* denotes the number of inbred lines belonging to each haplotype group. In the box plots, centre values are medians, and whiskers indicate variability outside the upper and lower quartiles. Statistical significance was determined using a two‐sided *t*‐test.

A SNP within GRMZM2G087806 residing on chromosome 9 was identified in our previous research and found to be significantly associated with drought tolerance (−Log_10_ *P* = 1.79 × 10^−6^) in maize seedlings (Wang *et al.*, 2016). Phylogenetic analysis of the encoded protein sequence indicated that its closest homologous gene in Arabidopsis is *TIP1*/*PAT24* (Figure S1). Thus, this gene was named *ZmTIP1*. To better understand how genetic variation in *ZmTIP1* contributes to maize drought tolerance, 166 diverse, maize inbred lines that were randomly selected from the original association population were re‐sequenced, including germplasm from temperate and tropical/subtropical (TST) regions. A 4.1‐kb genomic sequence containing *ZmTIP1* and spanning the 5′‐untranslated region (UTR) to 3′‐UTR region of the gene was analysed, and a total of 390 SNPs and InDels (MAF ≥ 0.05) were newly identified (Table S1). Variations upstream of the coding sequence and a non‐synonymous SNP50 (the 17^th^ serine changed into phenylalanine) were found to be the most significantly associated with the survival rate (SR) of maize seedlings subjected to a severe drought, as calculated using a mixed linear model that accounted for the effects of population structure and cryptic relatedness (−log_10_ *P> *5; Figure 1a). Interestingly, the majority of the variants in the *ZmTIP1* promoter were completely in linkage disequilibrium (LD, *r* ^2^ = 1) with SNP50, which mainly constituted two haplotypes (Hap1 and Hap2). Inbred lines carrying Hap2 had a statistically significant higher SR than those carrying Hap1, suggesting that Hap1 represents a drought‐sensitive allele, while Hap2 represents a drought‐tolerant allele (Figure 1b).

### *ZmTIP1* encodes a functional *S*‐acyltransferase

In yeast, *AKR1* encodes an *S*‐acyltransferase, and the *akr1* mutant exhibits elongated multinucleate cells and poor viability when grown at 30 °C and 37 °C (Roth *et al.*, 2002). Since the drought‐sensitive inbred line Mo17 carries Hap1, and the drought‐resistant inbred line CIMBL55 harbours Hap2, the CDS of *ZmTIP1* in Mo17 and CIMBL55, which differs in SNP50, were transformed into the *akr1* mutant. As a result, the characteristic growth defects of *akr1* were comparably complemented by each *ZmTIP1* CDS (Figure S2a). In Arabidopsis, mutation of *TIP1* alters plant development, especially root hair elongation but not initiation (Hemsley *et al.*, 2005). Both maize CDS, *ZmTIP1* ^Mo17^ and *ZmTIP1* ^CIMBL55^, were transformed into the Arabidopsis *tip1‐3* mutant, driven by the constitutive cauliflower mosaic virus (CaMV) 35S promoter. Both alleles of *ZmTIP1* restored normal root hair elongation in the short root hair mutant (Figure S2b,c). Collectively, these results indicate that *ZmTIP1* encodes a functional *S*‐acyltransferase and that the genetic variants in the coding region do not affect *ZmTIP1* protein function.

### Genetic variations in the *ZmTIP1* promoter are linked to drought tolerance

**Figure 2:**
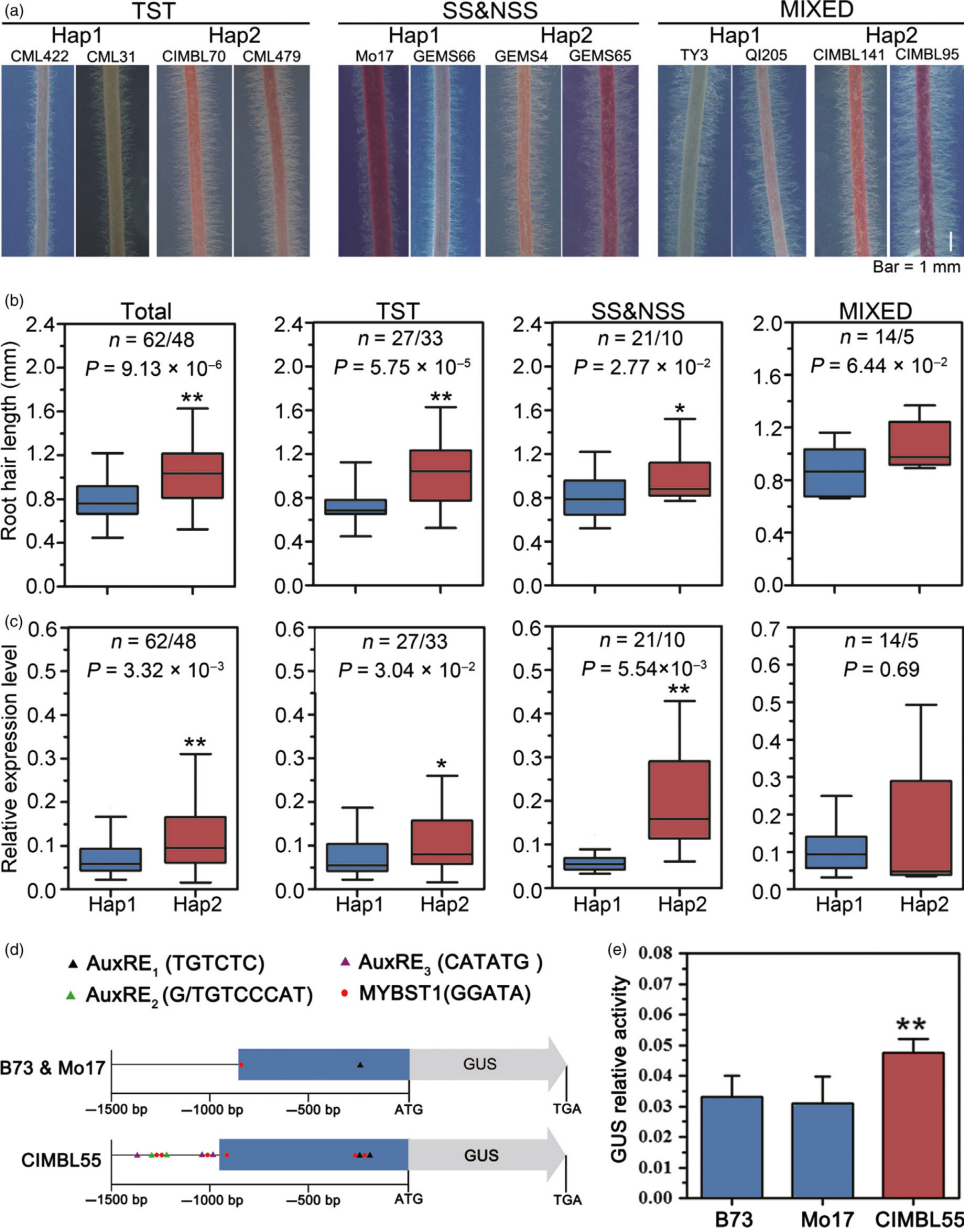
Genetic variations in the *ZmTIP1* promoter are linked to drought tolerance. (a) Representative photographs of root hairs of maize inbred lines from tropical/subtropical TST, temperate [including stiff stalk (SS) and non‐stiff stalk (NSS)] and mixed origins, grown on agar plates at seven days after germination. Bar = 1 mm. (b) Box plot distribution of root hair length in inbred lines possessing either the Hap1 or Hap2 allele. *n* is the number of analysed inbred lines of each haplotype. Statistical significance was determined by a two‐sided *t*‐test: *p < 0.05, **p < 0.01. (c) Box plot distribution of relative *ZmTIP1* mRNA levels in primary roots in different maize inbred lines under normal growth conditions. The *ZmUbi2* gene was used as an internal control. Statistical significance was determined by a two‐sided *t*‐test: *p < 0.05, **p < 0.01. (d) Schematic diagram of the 1.5‐kb promoters of *ZmTIP1* ^B73^, *ZmTIP1* ^Mo17^ and *ZmTIP1* ^CIMBL55^. Three different types of AuxREs are indicated by black, purple and green triangles. MybSt1 binding sites are indicated by red dots. (e) GUS enzyme activity driven by the 1.5‐kb promoter fragments of *ZmTIP1* ^B73^, *ZmTIP1* ^Mo17^ and *ZmTIP1* ^CIMBL55^. *Ubi:Luciferase* was co‐transfected and served as a positive control for transfection efficiency. The data are based on three independent biological replicates. Data represent the mean ± SD. Statistical significance was determined by a two‐sided *t*‐test: **p < 0.01.

Given the large number of significant genetic variants present in the promoter region of *ZmTIP1*, which mainly comprises two haplotypes, *ZmTIP1* expression and root hair length were comprehensively analysed in 110 maize inbred lines, which included 60 tropical/subtropical (TST) inbred lines, 31 temperate lines [including stiff stalk (SS) and non‐stiff stalk (NSS)] and 19 lines of mixed origin. In general, the results indicated that root hair length of the inbred lines carrying Hap2 was significantly longer than those carrying Hap1 (Figure 2a,b) and that *ZmTIP1* expression in Hap2 inbred lines was significantly higher than in Hap1 inbred lines (Figure 2c), while the difference in the root hair length and *ZmTIP1* expression was less obvious among the inbred lines of mixed origin (Figure 2). A genomic BAC library of CIMBL55 was screened to obtain the genomic sequence of *ZmTIP1^C^* ^IMBL55^. Notably, a significant number of sequence differences were identified upstream of *ZmTIP1* in Mo17 vs. CIMBL55; however, a good level of sequence synteny was identified between B73 and Mo17, two temperate inbred lines (Figure S3). Notably, the 1.5‐kb upstream sequences of *ZmTIP1* ^CIMBL55^ contain several types of AuxREs (auxin response elements), two copies of a typical TGTCTC sequence and two kinds of atypical sequences, G/TGTCCCAT (two copies) and CATATG (three copies), which were previously identified in the pea *PS*‐*IAA4/5* promoter and soya bean *SAUR 15A* promoter, respectively, and proposed to result in hypersensitive gene expression to auxin (Ballas *et al.*, 1993; Ulmasov *et al.*, 1995; Xu *et al.*, 1997) whereas B73 and Mo17 possess only one copy of a typical AuxRE, TGTCTC (Figure 2d). In addition, within the 1.5‐kb upstream fragment of *ZmTIP1* ^CIMBL55^ also possesses six copies of potato MybSt1 (Myb *Solanum tuberosum* 1) binding site (GGATA), which can induce fivefold to sevenfold higher levels of gene expression in response to the MybSt1 transcription factor (Baranowskij *et al.*, 1994). In contrast, the alleles of B73 and Mo17 only contain one copy of this sequence (Figure 2d). A close ortholog of *MybSt1* exists in the maize genome and also expresses in maize roots. Therefore, both fragments were cloned and placed in front of a *GUS* (*β‐glucuronidase*) reporter gene to compare their ability to promote gene expression. Results indicated that a higher level of GUS enzyme activity was detected using the *ZmTIP1* ^CIMBL55^ promoter compared to the *ZmTIP1* ^B73^ and *ZmTIP1* ^Mo17^ promoter (Figure 2e), indicating that sequence differences in the *ZmTIP1* promoter affected gene expression, which was potentially the basis for functional variation of *ZmTIP1* in the Hap 1 and Hap 2 haplotypes.

### Enhancing *ZmTIP1* expression increases root hair elongation and drought tolerance in Arabidopsis and maize

**Figure 3:**
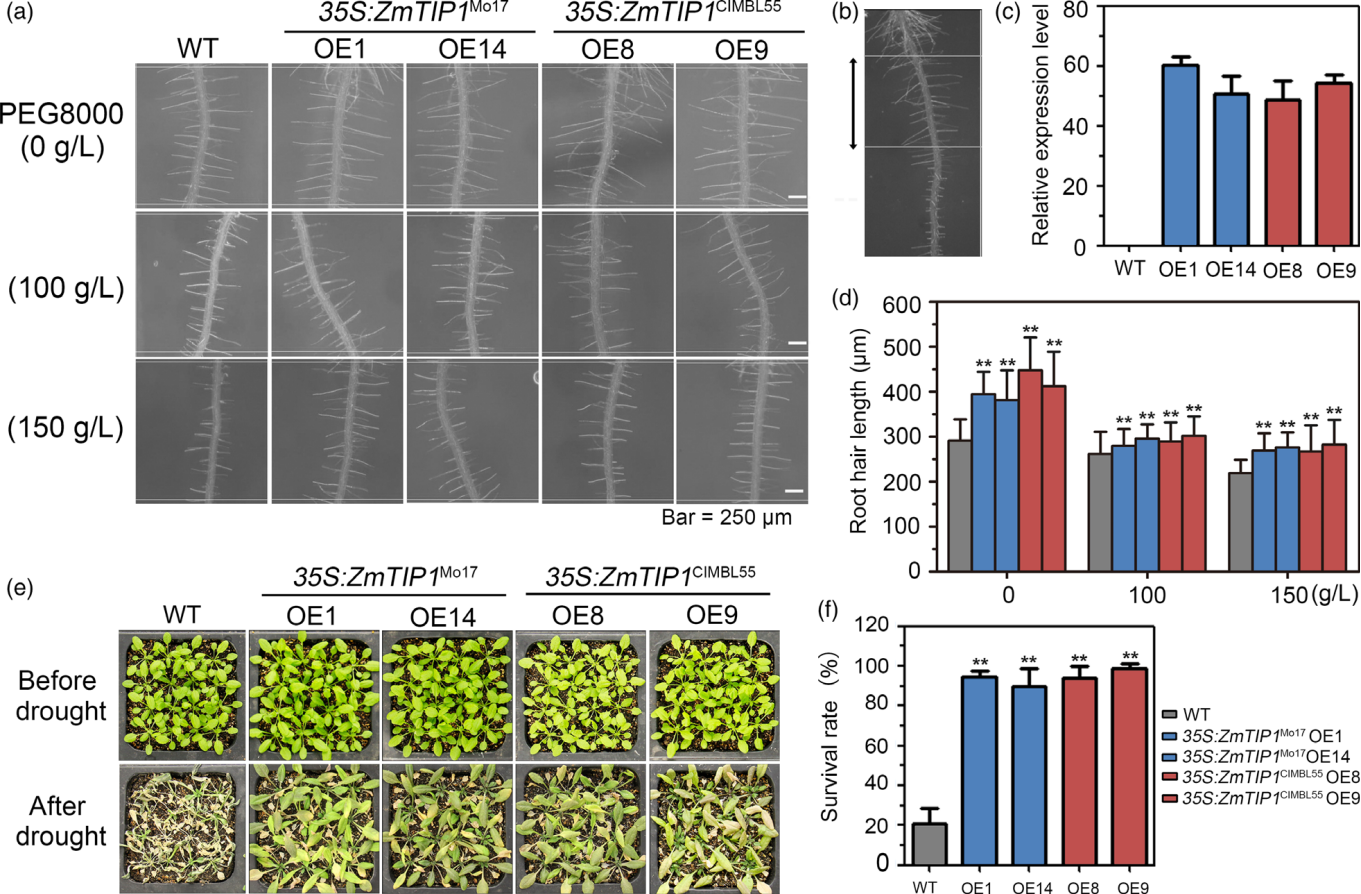
Root hair length and drought tolerance in *ZmTIP1* transgenic Arabidopsis. (a) Representative photograph of root hairs in five‐day‐old WT, *35S:ZmTIP1* ^Mo17^ and *35S:ZmTIP1* ^CIMBL55^ plants grown on half‐strength MS plates supplemented with PEG8000. Bar = 250 μm. (b) A representative photograph of Arabidopsis primary root hairs in plants grown on half‐strength MS plates for five days. The black arrows denote the region used for root hair length measurements in (a). (c) Transcript levels of *ZmTIP1* in WT and four independent *35S:ZmTIP1* transgenic Arabidopsis lines. (d) Average root hair length measured from approximately 600‐700 root hairs from at least 25 seedlings of each line. Statistical significance was determined by a two‐sided *t*‐test: **p < 0.01. (e) Representative photographs of homozygous T_3_ *35S:ZmTIP1* transgenic and WT plants subjected to a drought stress*.* A total of 192 seedlings were used for each genotype in three repeated experiments. The pictures were taken under well‐watered conditions and after thirteen days of no watering (drought treatment), followed by re‐watering for a period of 3 days. (f) Survival rate (SR) of *35S:ZmTIP1* transgenic and WT plants after drought treatment and recovery. Data represent the mean ± SD. Statistical significance was determined by a two‐sided *t*‐test: **p < 0.01.

**Figure 4:**
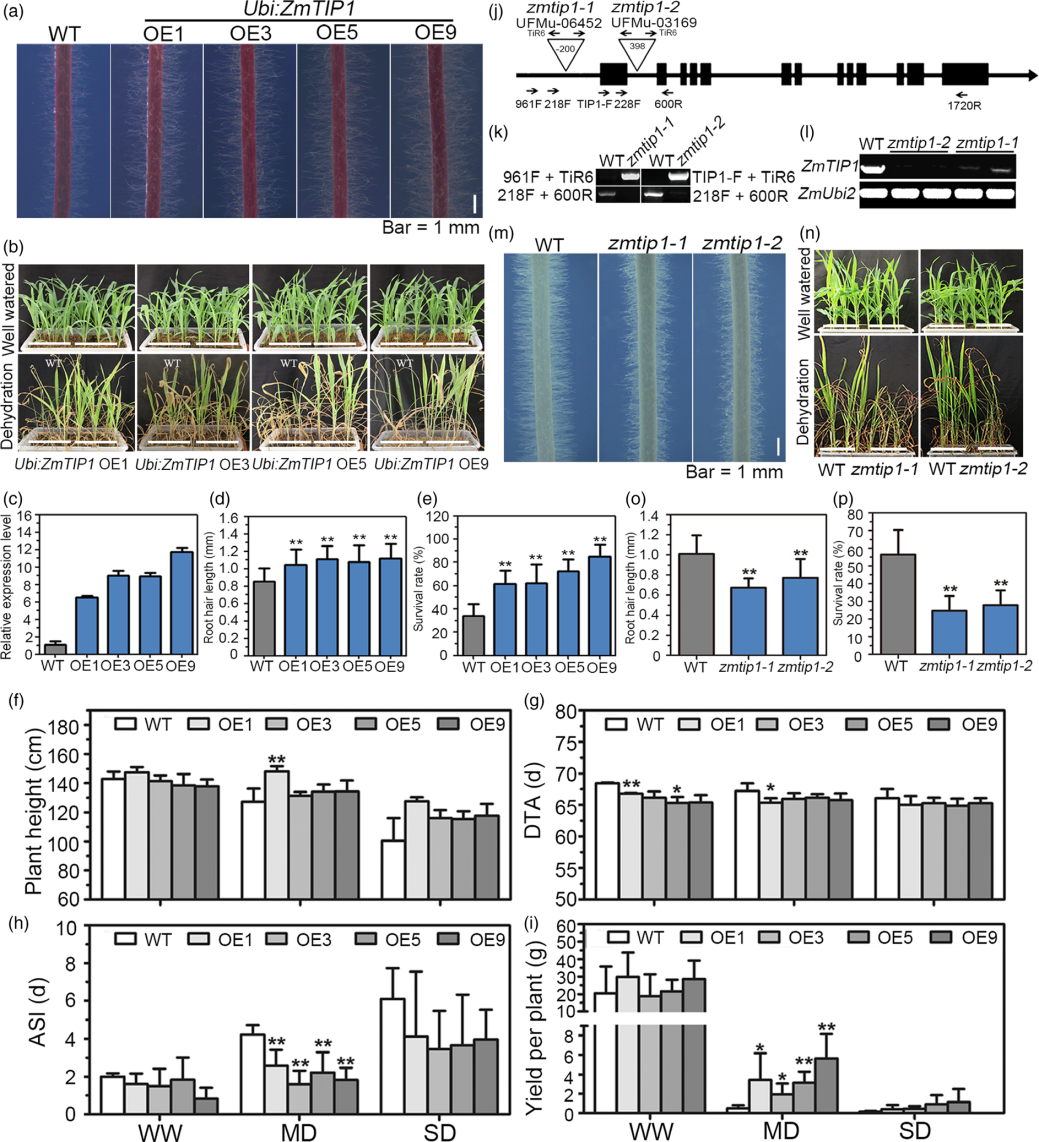
Analysis of root hair length and drought tolerance in *ZmTIP1* transgenic maize and *zmtip1* mutants. (a, m) Representative photograph of the root hairs of transgenic maize lines and *zmtip1* mutants grown on agar plates for seven days. Bar = 1 mm. (b, n) Drought tolerance of transgenic maize lines and *zmtip1* mutants. Photographs were taken before and after drought treatment followed by re‐watering for a period of 3 days. (c) Relative expression of *ZmTIP1* in WT and four independent *Ubi:ZmTIP1* transgenic lines under normal conditions. (d, o) Root hair length in primary roots of WT, *Ubi:ZmTIP1* transgenic maize, *zmtip1‐1* and *zmtip1‐2* seedlings. Data represent the mean ± SD of at least 25 plants. The experiment was conducted three times, and statistical significance was determined by a two‐sided *t*‐test: **p < 0.01. (e, p) Survival rate (SR) after drought treatment and recovery in WT, *Ubi:ZmTIP1* transgenic maize, *zmtip1‐1* and *zmtip1‐2* seedlings. Data represent the mean ± sd obtained from at least 90 seedlings in each line in three independent tests. Statistical significance was determined by a two‐sided *t*‐test: **p < 0.01. (f) Plant height, (g) days to anthesis (DTA), (h) the anthesis–silking interval (ASI), and (i) yield per plant in WT and the transgenic lines under well‐watered, moderate (MD) and severe drought (SD) conditions in the field. Data represent the mean ± SD of at least 50 plants for each line. Statistical significance was determined by a two‐sided *t*‐test: *p < 0.05, **p < 0.01. (j) Schematic structure of *ZmTIP1* and the positions of the Mu transposon insertion in *zmtip1‐1* and *zmtip1‐2*. Black boxes indicate exons, and lines indicate introns, upstream and downstream regions of *ZmTIP1*. Triangles represent the Mu transposon insertion. (k) Amplification of *ZmTIP1* from genomic DNA of WT, *zmtip1‐1* and *zmtip1‐2* plants. TiR6, TIP1‐F and 961 F primers were used for specific amplification of the Mu insertions, and 218 F and 600 R primers were used for *ZmTIP1* genomic sequence amplification. (l) Semi‐quantitative PCR analysis of *ZmTIP1* expression in the WT and mutant lines. Gene expression was determined using the 228 F and 1720 R primer pair. Two biological replicates are shown for each mutant. The *ZmUbi2* gene was used as an internal control.

Given that *ZmTIP1* may regulate root hair elongation and be associated with plant drought tolerance, *35S:ZmTIP1* ^Mo17^ and *35S:ZmTIP1* ^CIMBL55 ^transgenic Arabidopsis lines were generated. Two independent transgenic lines for each construct (*35S:ZmTIP1* ^Mo17^‐OE1 and OE14; *35S:ZmTIP1* ^CIMBL55^‐OE8 and OE9) were analysed. Root hair length in the transgenic plants was compared with the vector‐transformed plants (WT) in the presence or absence of PEG, a solute that can be used to stimulate the type of stress response imposed by a drying soil (Verslues *et al.*, 2006). All of the transgenic lines displayed increased root hair length in both normal and PEG growth conditions, although PEG stress clearly inhibited root hair elongation in all of the tested genotypes (Figure 3a‐d). Importantly, a greater survival rate (SR) was observed in all of the pot‐grown transgenic lines than in the WT plants (Figure 3e,f). Transgenic maize plants were generated using the strong constitutive promoter, *ZmUbi,* to determine whether enhancing *ZmTIP1* expression increases root hair elongation and drought tolerance in maize. Since the variants in the *ZmTIP1* CDS did not affect gene function, only the effect of the enhanced expression of the Mo17 CDS was examined. Root hair length and drought tolerance were examined in four independent lines. Results indicated that enhancing the expression of *ZmTIP1* significantly increased the hair length (Figure 4a,c,d). Side‐by‐side planting of transgenic and transgene‐negative siblings (WT) in soil‐containing pots also revealed improved drought tolerance in the transgenic lines (Figure 4b). The SR of the homozygous transgenic maize plants was approximately 65% under drought conditions, while the SR of WT plants was only 35% (Figure 4e). Transgenic lines were also compared with WT in replicated plots under a rain‐off shelter that were designed to simulate well‐watered (WW) and controlled drought conditions representing moderate (MD) and severe drought (SD). Under WW conditions, the phenotypic characteristics of the transgenic plants were almost identical to WT plants in terms of plant height, days to anthesis (DTA), anthesis–silking interval (ASI), and yield per plant, with the exception of precocious anthesis that was observed in the OE1 and OE5 transgenic lines (Figure 4f‐i). Under MD conditions, the transgenic lines exhibited greater grain yield than WT plants, which was most likely due to a reduced ASI in the transgenic lines, relative to WT plants (Figure 4h,i). No significant differences were observed between the transgenic lines and WT plants subjected to SD, under the tested conditions (Figure 4f‐i).

### *ZmTIP1* is essential for maize root hair elongation and drought tolerance

Two Mu transposon insertion lines, UFMu‐06452 (*zmtip1‐1*) and UFMu‐03169 (*zmtip1‐2*), were obtained from the public maize Mutator Line Stock Center (McCarty *et al.*, 2005) to further verify the involvement of *ZmTIP1* in root hair growth and drought tolerance*.* Locus‐specific PCR amplification was used to confirm that *zmtip1‐1* contained a Mu transposon insertion at 200‐bp upstream of the start codon and that *zmtip1‐2* had an insertion in the first intron (Figure 4j,k). Both lines were backcrossed to its wild‐type W22 for two generations, and the homozygous mutants were further analysed. Based on RNA samples isolated from two‐week‐old roots of W22 (WT) plants and the two homozygous mutants, *ZmTIP1* expression was extremely reduced in *zmtip1‐1*, and expression of the gene was faint in *zmtip1‐2* mutant (Figure 4l). Phenotypic tests demonstrated that the two mutants had significantly shorter root hairs and a lower SR compared to the WT (Figure 4m‐p). Collectively, these data indicate that *ZmTIP1* appears to play an essential role in root hair elongation and drought tolerance in maize.

### ZmTIP1 is localized on the plasma membrane (PM), prevacuolar compartments (PVC) and Golgi

**Figure 5:**
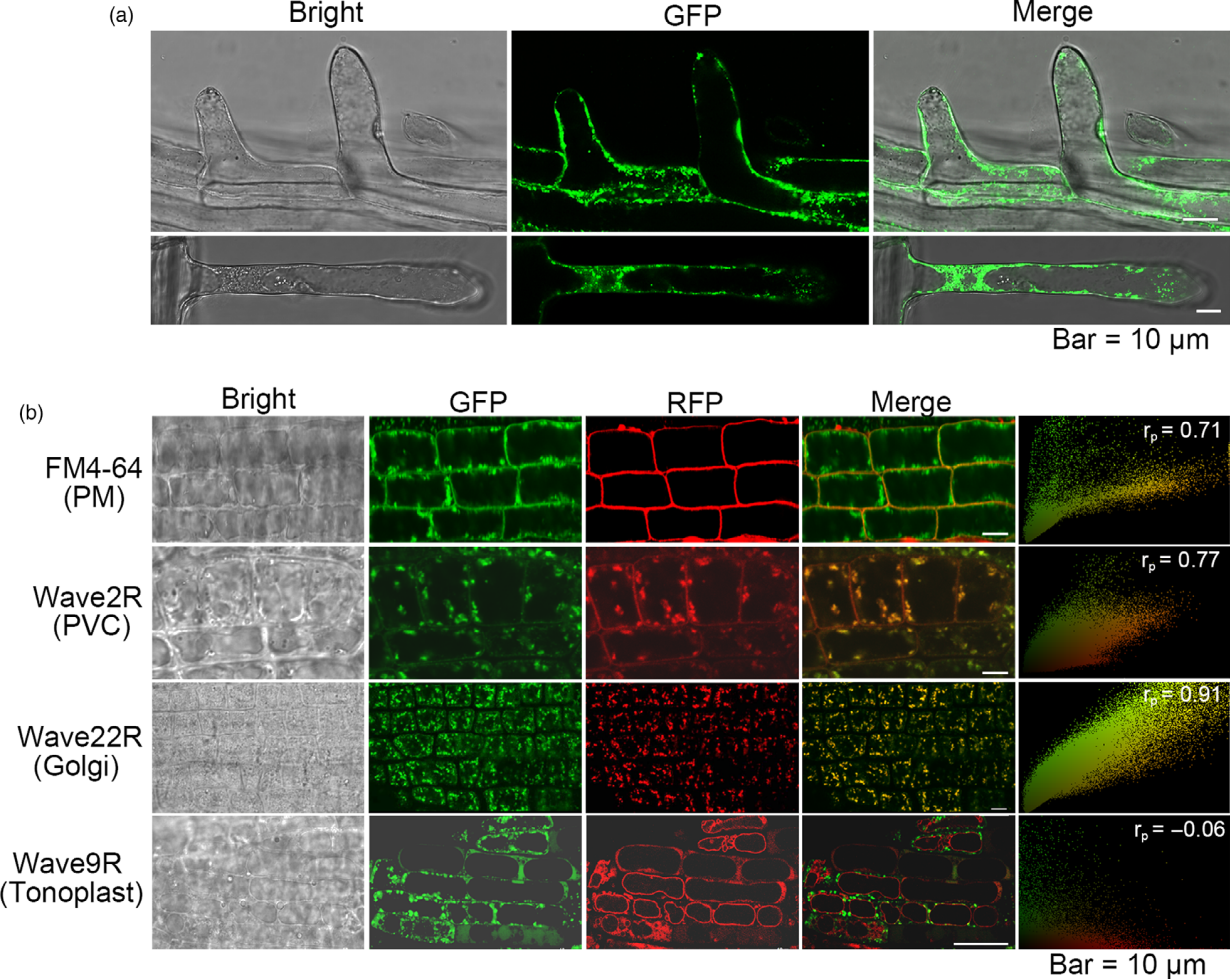
ZmTIP1 is localized on the plasma membrane (PM), prevacuolar compartment (PVC) and Golgi. (a) Confocal microscopy of transgenic Arabidopsis root hairs expressing *35S:ZmTIP1‐GFP*. Bar = 10 µm. (b) Roots of the *35S:ZmTIP1‐GFP* plants were stained with FM4‐64 for 5 min to reveal the plasma membrane (PM)*.* The *35S:ZmTIP1‐GFP* plants were crossed with Wave marker lines (Geldner et al., 2009) harbouring red fluorescence protein fused proteins targeting to prevacuolar compartments (PVC, Wave 2R), Golgi apparatus (Wave 22R) and tonoplast (Wave 9R), respectively. The F1 plants were observed under confocal microscope. Bar = 10 µm. Pearson’s correlation coefficient (*r*_p_) is shown at top right corner of each micrograph.

The subcellular location of ZmTIP1‐GFP was determined using confocal fluorescence microscopy to explore the biological function of ZmTIP1. ZmTIP1‐GFP signals were found on the PM and in the cytoplasm where they appeared as many spot‐like fluorescent foci in root hair cells (Figure 5a). The ZmTIP1‐GFP signal was observed to partially overlap with the red fluorescence of FM4‐64, a lipophilic fluorescent dye, after 5 min of staining, indicating that ZmTIP1 could localize to the PM. To better elucidate the cytosolic spot‐like signals, the *ZmTIP1‐GFP* lines were crossed with transgenic marker lines expressing red fluorescent protein (RFP)‐labelled endomembrane markers (Geldner *et al.*, 2009; Zhou *et al.*, 2013). A strong co‐localization was visualized in the PVC and Golgi apparatus (Figure 5b). ZmTIP1‐GFP, however, did not co‐localize with the tonoplast marker.

### ZmTIP1 mediates the ZmCPK9 PM association through *S*‐acylation

An affinity purification analysis was performed to identify *ZmTIP1*‐interacting proteins *in planta*. Total protein was extracted from 10‐d‐old *35S:ZmTIP1‐GFP* and *35S:GFP* (as the control) transgenic Arabidopsis. Proteins commonly detected in four replicate purifications, but not in the *35S:GFP*‐transformed plants, were regarded as potential ZmTIP1‐GFP‐interacting proteins. Several peptides representing CPK21 (Calcium‐dependent Protein Kinase 21, At4g04720) were resolved (Table S2), and a phylogenetic analysis indicated that its closest ortholog was ZmCPK9 in maize (Figure S4a, Ma *et al.*, 2013; Weckwerth *et al.*, 2015). *ZmCPK9* is highly expressed in roots and root hairs (Figure S4b, Saijo *et al.*, 1997). Interestingly, OsCPK2, an ortholog in rice, was reported to be palmitoylated at the N‐terminal MGSCCS motif, and the two cysteine residues were identified as the palmitoylation sites, which was determined to be essential for OsCPK2 PM association (Martin and Busoconi, 2000). Its modifier, however, remains unknown.

**Figure 6:**
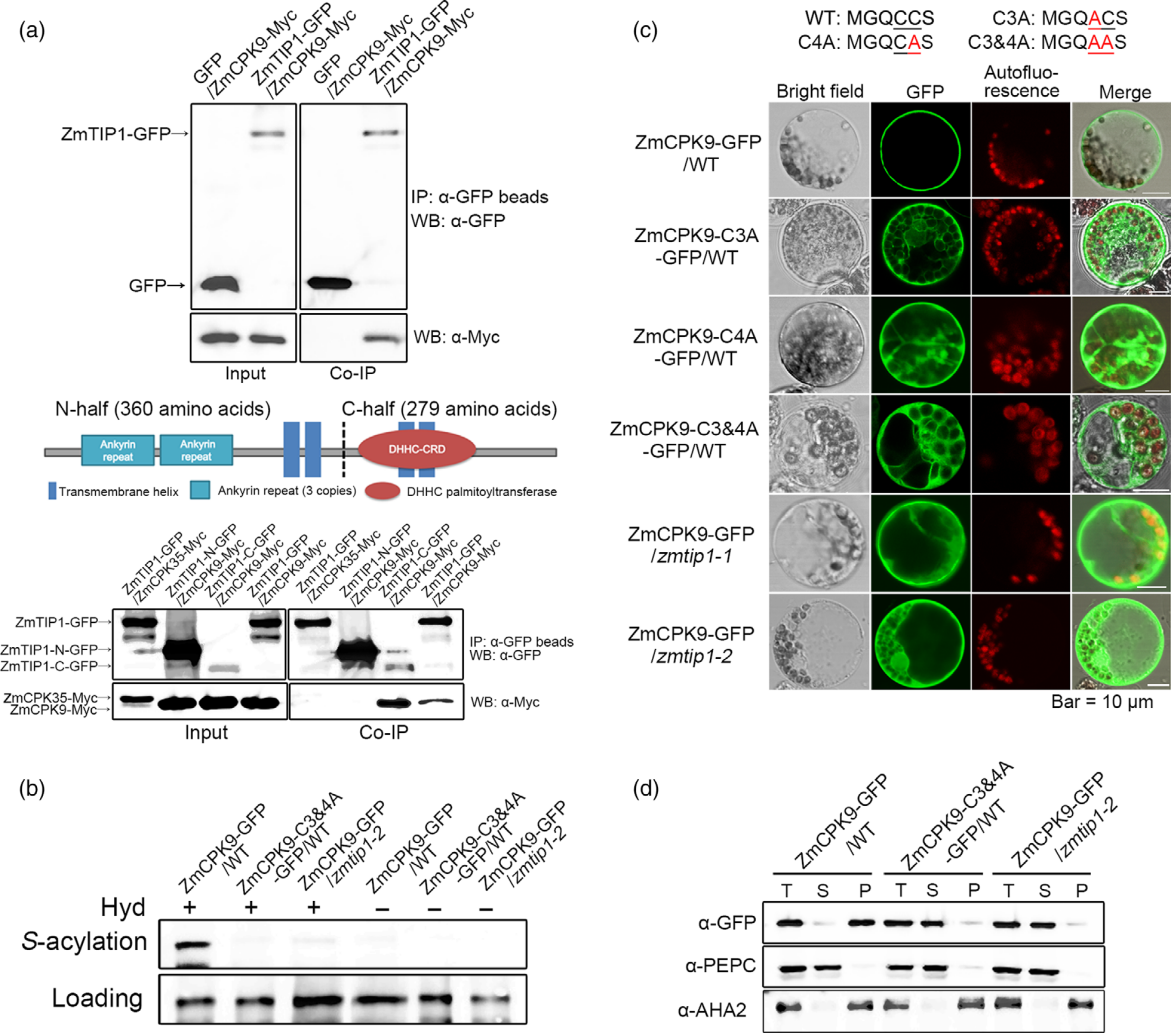
ZmTIP1 mediates ZmCPK9 PM association through *S*‐acylation. (a) Interaction of ZmTIP1 and ZmCPK9. Upper panel: co‐IP detection of the full ZmTIP1 protein with ZmCPK9 interaction. Middle panel: schematic diagram of ZmTIP1 protein structure. Lower panel: co‐IP detection of ZmTIP1 N‐half and C‐half interaction with ZmCPK9. ZmCPK35, a close homolog of ZmCPK9, was used in parallel as a negative control. Total proteins were extracted from protoplasts expressing GFP/ZmCPK9‐Myc, ZmTIP1‐GFP/ZmCPK9‐Myc, ZmTIP1‐GFP/ZmCPK35‐Myc, ZmTIP1‐N‐GFP/ZmCPK9‐Myc and ZmTIP1‐C‐GFP/ZmCPK9‐Myc. Input and co‐immunoprecipitated proteins were analysed using anti‐GFP and anti‐Myc antibodies, respectively. (b) ZmTIP1‐dependent *S‐*acylation of ZmCPK9 as evidenced in the biotin switch assay. Samples were treated with or without a thioester cleavage reagent, hydroxylamine (Hyd^+^ or Hyd^‐^). Upper panel labelled with ‘*S*‐acylation’ indicates *S*‐acylated ZmCPK9‐GFP. Lower panel labelled with ‘loading’ shows that equal amounts of protein were loaded onto the NeutrAvidin beads. (c) Confocal microscopy images revealing the subcellular localization of ZmCPK9‐GFP, ZmCPK9‐C3A‐GFP, ZmCPK9‐C4A‐GFP and ZmCPK9‐C3&4A‐GFP fusion proteins in WT, *zmtip1‐1* and *zmtip1‐2* maize mesophyll protoplasts. Bar = 10 μm. (d) Western blot analysis of ZmCPK9‐GFP and ZmCPK9‐C3&4A‐GFP localized in different cell fractions. The total cell lysate (T), cytosolic soluble fraction (S) and pellet of the membrane fraction (P) were prepared from WT and *zmtip1‐2* maize mesophyll protoplasts. The immunoblots utilized anti‐GFP, anti‐AHA2 (a plasma membrane ATPase, as a marker of membrane fraction) and anti‐PEPC (phosphoenolpyruvate carboxylase, as a marker of cytosolic protein) antibodies. Results shown are representative of data obtained from three biological replicates.

Co‐immunoprecipitation (co‐IP) assays using maize protoplasts expressing *Ubi:ZmTIP1‐GFP* and *Ubi:ZmCPK9‐Myc* verified the interaction of ZmTIP1 and ZmCPK9 *in vivo* (Figure 6a). Since the ZmTIP1 protein is predicted to contain four transmembrane motifs, ankyrin repeats and a DHHC domain, we wanted to determine which part of the protein might substantially mediate the interaction with ZmCPK9. Results indicated that although the N‐terminal half of ZmTIP1 could be highly co‐expressed with ZmCPK9‐Myc in maize protoplasts, it was barely capable of pulling down ZmCPK9. In contrast, the C‐terminal half of ZmTIP1, containing the DHHC domain, could effectively immunoprecipitate with ZmCPK9, even though it was less expressed. ZmCPK35, a close homolog of ZmCPK9, was tested in parallel to rule out the possibility of nonspecific detection (Figure 6a, Figure S4a). Taken together, these results indicate that the C‐terminal half of ZmTIP1, which is predicted to possess *S*‐acylation enzymatic activity, preferably interacts with the substrate protein ZmCPK9. The biotin switch assay was previously developed to test whether proteins are *S*‐acylated‐modified. In this assay, *S*‐acyl group cleavage reagent, hydroxylamine (Hyd), liberates free sulfhydryls that are subsequently labelled with sulfhydryl reactive biotin and bound by NeutrAvidin beads. Non‐*S*‐acylated proteins or proteins without Hyd treatment cannot be labelled with biotin and are thus undetectable after NeutrAvidin bead purification (Hemsley *et al.*, 2008). We tested whether or not ZmCPK9 could be *S*‐acylated by ZmTIP1 in maize protoplasts. Results indicated that ZmCPK9‐GFP receiving the Hyd treatment could bind to NeutrAvidin beads and be detected by anti‐GFP (Figure 6b), suggesting that ZmCPK9 is *S*‐acylated in WT protoplasts. However, the signal was substantially reduced when ZmCPK9‐GFP was examined in *zmtip1‐2* protoplasts. No signal was detected from the non‐*S*‐acylatable ZmCPK9‐C3&4A mutant protein when it was similarly expressed in WT protoplasts.

Inspection of the ZmCPK9 sequence revealed that the protein N‐terminal contains a similar MGQCCS motif, indicating that it might be a potential modification site (Figure S4a). Therefore, three non‐*S*‐acylatable variants were generated with alanine (A) substitutions at the 3rd C (C3A), at the 4th C (C4A) and at both cysteines (C3&4A), when the first methionine (M) is not counted (Figure 6c). The subcellular localization of ZmCPK9‐GFP was initially examined in WT maize protoplasts using confocal microscopy. ZmCPK9‐GFP was predominantly detected on PM, with only a faint signal in the cytoplasm (Figure 6c). In contrast, a more prominent GFP signal was observed in the cytoplasm of both point mutants (C3A and C4A), while the signal in the C3&4A mutant was mainly observed in the cytoplasm. The localization of ZmCPK9‐GFP in *zmtip1‐1* and *zmtip1‐2* protoplasts was then examined, and a similar pattern was observed as that of the non‐*S‐*acylatable mutant proteins. Membrane fractionation and immunoblot analyses with anti‐GFP antibodies were performed to confirm the microscopy observations. Results consistently indicated that ZmCPK9‐GFP was mainly detected in the precipitation of the membrane fraction, while the C3&4A mutant was readily detected in the soluble fraction of cytosol (Figure 6d). Additionally, ZmCPK9‐GFP was abnormally detected in the cytosol in the *zmtip1‐2* mutant, similar to the C3&4A mutant in WT protoplasts.

Collectively, the results suggest that ZmCPK9 serves as a target substrate of ZmTIP1 for *S*‐acylation modification *in vivo*. These data strongly indicate that ZmTIP1 mediates ZmCPK9 *S*‐acylation, which facilitates the association of ZmCPK9 with the PM.

## Discussion

Root hair cells play an important role in increasing the effective area of roots involved in water uptake and in the interaction of roots with rhizosphere organisms. Based on the previous results of a GWAS analysis, we conducted a comprehensive study on a candidate gene *ZmTIP1* whose sequence variation was significantly associated with drought tolerance in maize seedlings. Re‐sequencing a 4.1‐kb genomic fragment containing *ZmTIP1* in 166 maize inbred lines of different geographic origins and climates revealed significant genetic variations in the promoter region of *ZmTIP1*, together with a non‐synonymous variant in CDS, SNP50, due to a complete LD in these variations (Figure 1a). Further analysis indicated that the amino acid substitution brought by SNP50 had no obvious effect on protein function, since both alleles were able to rescue a mutant phenotype in yeast and Arabidopsis (Figure S2). This suggested that the significance of SNP50 was probably due to a linkage effect in the promoter. Although a number of variations were identified in the *ZmTIP1* promoter, they mainly comprised two haplotypes, and the maize inbred lines harbouring Hap2 exhibited a higher SR and longer root hairs along with higher *ZmTIP1* expression, relative to inbred lines carrying Hap1 (Figures 1 and 2). In addition, GUS activity driven by the 1.5‐kb promoter fragment cloned from *ZmTIP1* ^CIMBL55^ was significantly higher than that driven by *ZmTIP1* ^Mo17^ promoter fragment (Figure 2e). A sequence analysis of the region upstream from the start codon of *ZmTIP1* revealed significant variation among three representative maize genotypes. TST germplasm, CIMBL55, displayed especially large differences from two temperate genotypes, B73 and Mo17. A *Gypsy*‐176 retrotransposon insertion is present in the −1.5 kb to −0.9 region of B73 and Mo17 (Figure S3). In contrast, a *hAT*‐25 DNA transposon and a *Gypsy*‐68 retrotransposon are present in CIMBL55. Additional AuxREs and Mybst1 sequences were identified within the 1.5‐kb upstream fragment of *ZmTIP1* ^CIMBL55^ compared with those of *ZmTIP1* ^B73^ and *ZmTIP1* ^Mo17^ alleles. Although the actual sequence alteration that induces greater expression of Hap2 in roots requires further study, our data suggest that variants in the promoter region of *ZmTIP1* ^CIMBL55^ confer higher promoter activity.

Results of the present study highlight the positive role that *ZmTIP1* plays in regulating root hair elongation and drought resistance in maize. Importantly, there is some controversy on the necessity of root hairs in water absorption since some root hairless mutants are viable without exhibiting water deficit symptoms, indicating that root hairs may not be absolutely necessary for adequate water uptake. Most researchers, however, have indicated a positive role for root hairs in the efficient uptake of water nutrients, especially under drought stress. A comparison of the relationship between transpiration rate and xylem suction indicated that the capacity to take up water from moderately dry soils is substantially reduced in a root hairless mutant relative to wild‐type plants (Carminati *et al.*, 2017). The root hairless transgenic line, NR23, overexpressing the N‐terminal 23‐residue of a plasma membrane‐associated Ca^2+^‐BINDING PROTEIN2 under the control of a root hair‐specific *EXPANSIN7* promoter exhibited a 47% reduction in water uptake, as well as an increased sensitivity to drought stress compared to wild‐type plants (Tanaka *et al.*, 2014). Notably, QTLs for root hair length located on chromosomes 2A and 6A co‐localize with QTLs for yield components in wheat, suggesting that longer root hairs may contribute to yield potential (Horn *et al.*, 2016). In this study, we reported that ZmTIP1 functions in root hair development by mediating ZmCPK9 *S*‐acylation and PM association, which may contribute to efficient water uptake and drought tolerance. Collectively, the data indicate that enhancing root hair function may represent a potential strategy for improving drought tolerance and perhaps crop productivity.

There are 38 genes encoding predicted *S*‐acyltransferases in the maize genome (Yuan *et al.*, 2013). Direct experimental evidence on individual PATs and their target substrate protein is still very much lacking. Unlike transmembrane proteins, attachment of peripheral proteins to membranes can be brought about through lipid modifications, including *N*‐myristoylation and/or *S*‐acylation (Greaves and Chamberlain, 2007). Some evidence has been provided, indicating that N‐terminal protein myristoylation at glycine and/or *S*‐acylation of an adjacent cysteine is essential for protein PM targeting in plants (Gutermuth *et al.*, 2013; Hemsley *et al.*, 2013a; Liu, *et al.*, 2013a; Saito *et al.*, 2018). In the present research, although the protein affinity purification was carried out in a heterogeneous plant system, we successfully identified ZmCPK9 as a substrate protein for ZmTIP1 in maize, suggesting that a conserved mechanism may exist in different plant species. We verified that the 3^rd^ cysteine and 4^th^ cysteine in the N‐terminal MGSCCS motif of ZmCPK9 are *S*‐acylation‐modified, which was necessary for the ZmCPK9 PM association to occur in wild‐type maize protoplasts (Figure 6a,b). In contrast, in *zmtip1‐1* and *zmtip1‐2*, ZmCPK9 were abnormally retained in the cytosol, suggesting that ZmTIP1 is essential for the modification of ZmCPK9 and PM association. By using co‐IP and biotin switch analyses, ZmCPK9 was successfully identified as a direct substrate protein of ZmTIP1 in maize (Figure 6c,d). CPKs as a type of calcium sensor, which contain a variable N‐terminal region, a kinase catalytic domain and an autoinhibitory junction domain, are followed by a calmodulin‐like domain (CaM‐LD) (Hrabak *et al.*, 2003). CPKs were identified to be involved in various abiotic stress signalling pathways in planta. The PM association of ZmCPK32 is essential for polar cell growth, as expressing the cytosol‐localized kinase displayed reduced pollen tube growth (Li *et al.*, 2018). In ABA signalling, AtCPK21 and AtCPK23 phosphorylate and activate SLAC1 (Slow Anion Channel‐Associated 1) to promote stomatal closure (Geiger *et al.*, 2010; Ma and Wu, 2007). AtCPK9 was reported localized on cell membrane and function in stomatal movement in response to ABA treatment (Chen *et al.*, 2019). Whether AtCPK9 plays a role in root hair elongation needs further investigations. Based on the data obtained, we propose that ZmTIP1 enhances root hair elongation most likely by mediating the ZmCPK9 PM association. During root hair elongation, a high concentration of Ca^2+^ is present at the apex of the root hair (Libault *et al.*, 2010). Recently, it is reported that several cyclic nucleotide‐gated channels (CNGCs) function as Ca^2+^ channels, which are essential for constitutive root hair growth in Arabidopsis (Tan *et al.*, 2019). Whether the PM‐associated ZmCPK9 is to stimulate Ca^2+^ or anion channels to promote tip growth is not fully known. In addition, the increased cytosolic Ca^2+^ can activate NADPH oxidase activity in root hairs to generate rapid ROS burst, which is required for polar cell elongation (Kimura *et al.*, 2012; Nestler *et al.*, 2014). It is also possible that ZmCPK9 facilitates root hair elongation through activating NADPH oxidase on the PM. Although hundreds of proteins have been postulated to be modified by *S*‐acylation in plants, their modifiers are largely remained to be identified. Further research is required to document PAT/substrate selectivity and to understand the biological function of individual PATs. Collectively, our findings provide a new genetic strategy to improve plant root hair length and drought tolerance, and also increase our knowledge on ZmTIP1‐mediated ZmCPK9 *S*‐acylation.

## Experimental procedures

### Association analysis of *ZmTIP1* with drought tolerance

The re‐sequencing and association analyses of *ZmTIP1* were performed using 166 maize inbred lines, which were representative of the whole association‐mapping panel composed of 367 diverse maize inbred lines. The *ZmTIP1* promoter (~1.5 kb), coding regions (without introns) and 3´‐UTR sequences were amplified by PCR and sequenced. Nucleotide polymorphisms, including SNPs and InDels, were identified, and their association with the survival rates of maize seedlings subjected to drought stress was calculated by TASSEL 5.0 (Bradbury *et al.*, 2007) using a standard mixed linear method (MLM, with MAF ≥ 0.05), in which the population structure (Q) and kinship (K) were estimated as previously described (Liu, *et al.*, 2013b). The primers used in the current study are listed in Table S3.

### Complementation of *akr1* defects with *ZmTIP1*

The *akr1* yeast culture was obtained from EURO‐Scarf (http://www.uni-frankfurt.de/fb15/mikro/euroscarf/). The full‐length coding regions (CDS) of *ZmTIP1* amplified from maize inbred lines Mo17 (Hap1) and CIMBL55 (Hap2) were cloned into pYES2/NT vector (Invitrogen) under the GAL1 promoter using *Hind* III and *BamH* I sites. The constructed plasmids were transformed into *akr1*, and AH109 and *akr1* were transformed with the pYES2/NT vector as positive and negative controls. Cultures of each genotype were grown in selective liquid medium at 30 °C and 37 °C. Cell morphology was observed using a Nikon 80i light microscope. Three replicates were evaluated per genotype using independent transformants.

### Arabidopsis transformation and phenotypic analyses

The CDS of *ZmTIP1* in Mo17 and CIMBL55 were amplified and inserted into pGreenII vector (Qin *et al.*, 2008) under the CaMV 35S promoter using *Sma* I and *Sac* I sites. The constructed plasmid was transformed into *Agrobacterium tumefaciens* GV3101 containing the pSoup helper plasmid. *Arabidopsis thaliana* ecotype Col‐0 and mutant *tip1‐3* were transformed using the *Agrobacterium*‐mediated method. Homozygous T3 seedlings were identified by kanamycin‐based selection and used for subsequent phenotypic analysis. *ZmTIP1* gene expression in transgenics was determined by RT‐qPCR, in which *Actin2* was used as an internal control for standardization. The drought tolerance test was performed as described (Ding *et al.*, 2015). For root hair analyses conducted in Arabidopsis*,* seeds were sown in regular ½MS plates with 0.5% sucrose and ½MS plates supplemented with 0, 100 or 150 g/L PEG8000. PEG‐infused plates were prepared as previously described (Verslues *et al.*, 2006). A minimum of 600–700 root hairs from 25 plants for each genetic background were evaluated in each experiment. Quantification of root hair length was conducted from captured images using ImageJ. The experiment was repeated three times.

### Affinity purification of *ZmTIP1*‐interacting proteins

Transgenic Arabidopsis were generated by insertion of *35S:ZmTIP1‐GFP* constructs. The *ZmTIP1* CDS was inserted into pGreenII‐GFP vector using *Sma* I and *Sac* I sites (Ding *et al.*, 2015). Seedlings of ten‐day‐old T3 transgenic lines were used for further analysis. Affinity purification was performed according to Ding *et al *(2015). Proteins identified in the *35S:ZmTIP‐GFP* but not in the *35S:GFP* samples were considered as putative Z*mTIP1‐*interacting proteins.

### Confocal microscopy analysis of the ZmTIP1‐GFP subcellular localization

Root hairs of five‐day‐old T3 seedlings grown on vertical MS agar plates were observed and imaged using a fluorescence microscope (Leica TCS SP5) to determine the subcellular localization of *ZmTIP1*. Primary roots of five‐day‐old vertically grown *35S:ZmTIP1‐GFP* were stained in FM4‐64 (4 μm) in 0.5 × MS for 5 min, rinsed 3 times in 0.5 × MS and then imaged with an Olympus FV 1000 MPE (615 nm for FM4‐64) laser scanning confocal microscope. Additionally, *35S:ZmTIP1‐GFP* were crossed with RFP‐labelled WAVE 2R, Wave 9R and WAVE 22R plants in a Col‐0 background. The five‐day‐old F1 seedlings were grown on vertical MS agar plates and observed with the laser scanning confocal microscope (Olympus FV 1000 MPE, 488 nm for GFP and 543 nm for RFP). Images were further analysed with ImageJ software to acquire a linear Pearson correlation coefficient (r_p_) (French *et al.*, 2008).

### Maize protoplast transfection and cell fraction preparation

The CDS of ZmCPK9 and its mutants, ZmCPK9‐C3A, ZmCPK9‐C4A and ZmCPK9‐C3&4A, were cloned into pSBII‐Ubi:GFP vector by *Sma* I site for the subcellular localization analysis. Protoplasts were isolated from etiolated seedlings of maize inbred lines (W22, *zmtip1‐1* and *zmtip1‐2*) as previously described (Yoo *et al.*, 2007). After a 14‐h incubation period, transformed protoplasts were observed with a confocal microscope (Leica TCS SP5) and images were collected. Cell fractionation assays were performed as described (Liu *et al.*, 2017). Briefly, 700 μl of a protoplast suspension (10^6^ per ml) was transfected with 70 μg of DNA and incubated at 22 °C for 14 h. The protoplasts were collected and extracted in 700 μl of extraction buffer (50 mm Tris pH 8.0, 2 mm EDTA, 20% glycerol, 1 mm DTT, 0.1% Triton X‐100, and 1 × protease inhibitor cocktail). The samples were then centrifuged at 4 °C, 5000 g for 5 min to remove debris. Fifty μl of the supernatant was saved for total protein (T). The subcellular fractionation was performed by ultracentrifugation of the homogenate for 1 h at 4 °C, 100 000 g to obtain a soluble fraction (S) and a pellet (P). Anti‐PEPC (Agrisera, Cat#AS09458) and anti‐AHA2 antibodies (Yang *et al.*, 2010) were used as cytosolic and membrane fraction markers, respectively. The pellet (membrane fraction) was solubilized in 650 μL extraction buffer. For Western blotting, total proteins (T) and soluble fractions (S) were 1 × concentrated relative to the membrane protein extracts.

### Co‐immunoprecipitation assays

The full‐length, N‐terminal and C‐terminal half of *ZmTIP1* CDS were cloned into pGreenII‐Ubi‐GFP vector using the *BamH* I site. The CDS of ZmCPK9‐Myc and ZmCPK35‐Myc with a stop codon were cloned into pGreenII‐Ubi‐GFP vector using *Sma* I and *Not* I sites, resulting in the removal of GFP from the vector. The co‐immunoprecipitation assays were performed as described by Ding *et al. *(2015). Briefly, 1 mL of protoplasts transformed with different combinations of plasmids was used to extract total protein and immunoprecipitated with anti‐GFP mAb‐Magnetic Beads (MBL). The immunoprecipitates were detected with anti‐GFP (Abmart, Cat#M20004S) and anti‐Myc antibody (Sigma‐Aldrich, Cat#M4439, Shanghai, China).

### *S*‐acylation assay of ZmCPK9

The CDS of ZmCPK9 and the C3&4A mutant were cloned into pGreenII‐Ubi‐GFP vector using the *BamH* I site. Protoplasts were isolated from 14‐day‐old etiolated W22 and *zmtip1‐2* seedlings. Typically, 200 μL of a protoplast suspension (10^6^ cells per ml) was transfected with 15 μg plasmid, and a total of 1.2 mL of protoplasts of each sample was transfected in total. The biotin switch assay was conducted as described by Hemsley *et al. *(2008). Samples were analysed by SDS‐PAGE and Western blotting with anti‐GFP antibody.

### Transgenic maize construction and maintenance of *zmtip1* mutants

The *Ubi:ZmTIP1* expression cassette was delivered into the A188 maize inbred line as described by Ishida *et al. *(2007). Transgenic plants were grown in a greenhouse under a 16‐h/8‐h light/dark cycle. The positive transgenic plants of each generation were identified using *ZmTIP1*‐specific PCR analysis. The expression of *ZmTIP1* in transgenic plants was determined by RT‐qPCR, in which *ZmUbi2* was used as an internal control for standardization. Four independent homozygous lines were selected for further analysis. Seeds of *zmtip1‐1* (UFMu‐06452) and *zmtip1‐2* (UFMu‐03169) were obtained from the Maize Genetics Cooperation Stock Center (https://www.maizegdb.org/gbrowse). The *ZmTIP1* of these lines was genotyped, and heterozygous plants were successively backcrossed with W22 for two generations. The BC2F1 plants were self‐pollinated, and the homozygous mutants were used in subsequent analyses.

### Determination of root hair length and drought tolerance of maize seedlings

Maize seeds were sterilized in 10% (v/v) H_2_O_2_ for 30 min, washed with distilled water three times and soaked in saturated CaSO_4_ solution for 8 h. The seeds were then placed between two sheets of wet filter paper and germinated in the dark at 24 °C. When the primary roots were approximately 2 cm long, uniform seedlings were carefully transferred into 25‐cm vertical agar plates for four days of continued growth at 24–26 °C and a 16‐h /8‐h light/dark photoperiod. The images were captured with an Olympus fluorescence microscope (SEX16). A minimum of 700–800 root hairs in seven‐day‐old primary roots of 25 plants were analysed in each experiment. Quantification of root hair length was assessed using ImageJ software. The experiment was repeated three times. For the drought tolerance assay, transgenic‐positive and WT plants were planted side by side in 2 kg of soil (top soil, vermiculite and turf in a ratio of 1:1:1). A drought treatment was administered to the soil‐grown plants at the three‐leaf stage by withholding water. Significant differences in wilting were observed after approximately 25 days, and watering was then resumed to allow plants to recover. The number of surviving plants was recorded 3 days after re‐watering was commenced. At least 30 plants of each line were compared in each test, and statistical analyses were based on data obtained from at least three independent experiments.

### *ZmTIP1* promoter activity assay

The sequence analysis of the *ZmTIP1* promoter was conducted using the RepeatMasker Web Server (http://repeatmasker.org/cgi-bin/WEBRepeatMasker) and New PLACE (https://www.dna.affrc.go.jp/PLACE/?action=newplace). The 1.5‐kb fragments of the *ZmTIP1* promoter from the Mo17 and CIMBL55 inbred lines were cloned into the plant transient expression vector pIG46 using the *Hind* III site in front of the minimum 35S CaMV promoter. Protoplast was isolated from 14‐day‐old etiolated seedlings of B73 inbred line. For the GUS assay, Luciferase was co‐transfected and used as a reference for the analysis. The GUS activity and LUC activity were measured as previously described (Yoo *et al.*, 2007). The data were obtained from three biological replicates.

### Evaluation of drought tolerance under field conditions

Drought tolerance of WT (A188) and four independent *Ubi:ZmTIP1* transgenic plants were determined under field conditions during the summer of 2017 in a rain‐off shelter in Beijing, China (39°98′N, 116°21′E). The average temperature during the growing season was 25.1 °C. Seeds of all the lines were planted on 2 May 2017 at a density of 58,000 plants/hectare and harvested on 15 September 2017. Three replicate plots were utilized for well‐watered, moderate and severe drought (WW, MD and SD, respectively) treatments. The WW plots were irrigated with an adequate supply of water during the entire growing season, taking into account natural episodes of rainfall. Watering was stopped in the drought treatment plots at 21 d after sowing. Additional watering was provided to plants in the MD plots at the tasselling stage. Days to anthesis (DTA) was assessed for plants in all of the plots as the number of days from sowing to anther extrusion in the 5 cm tassel axis. Days to silk (DTS) was assessed as the number of days from sowing until 1 cm of silk was visible. The anthesis–silking interval (ASI) was calculated as the difference between DTA and DTS. All of the ears were naturally dried after harvest until the seed moisture content was approximately 11–14%, as measured by a PM‐8188‐A (Kett) moisture analyser. Yields (seed weight) per plant were calculated.

## Conflict of interest

The authors declare no conflict of interest.

## Author contributions

X.Z. performed all the experiments and drafted the manuscript. Y.M. and S.L. helped with the yield test of transgenic maize in fields. H.M. assisted in re‐sequencing analysis of *ZmTIP1.* L.C. assisted in the generation of transgenic maize lines. F.Q. conceived and provided advice on all of the experiments and revised the manuscript.

## Supporting information


**Figure S1** Phylogenetic analysis of PAT proteins in maize, rice, sorghum, and *Arabidopsis*.

**Figure S2** *ZmTIP1* encodes a functional S‐acyltransferase.

**Figure S3** Comparison of the *ZmTIP1* promoter in B73, Mo17, and CIMBL55 inbred lines of maize.

**Figure S4** Phylogenetic analysis of ZmCPK9 and its relative expression in root hairs.

**Table S1** Variations in the *ZmTIP1* genomic region and their association with drought tolerance in maize.

**Table S2** List of proteins identified by mass spectrometry analysis.

**Table S3** PCR primer sequences used in the presented research.
