# Supplementary figures and images for: Genetic variation in ZmTIP1 contributes to root hair elongation and drought tolerance in maize
Source: Plant Biotechnol J. 2019 Nov 19;18(5):1271–83. doi: 10.1111/pbi.13290 (PMC7152618; doi:10.1111/pbi.13290)

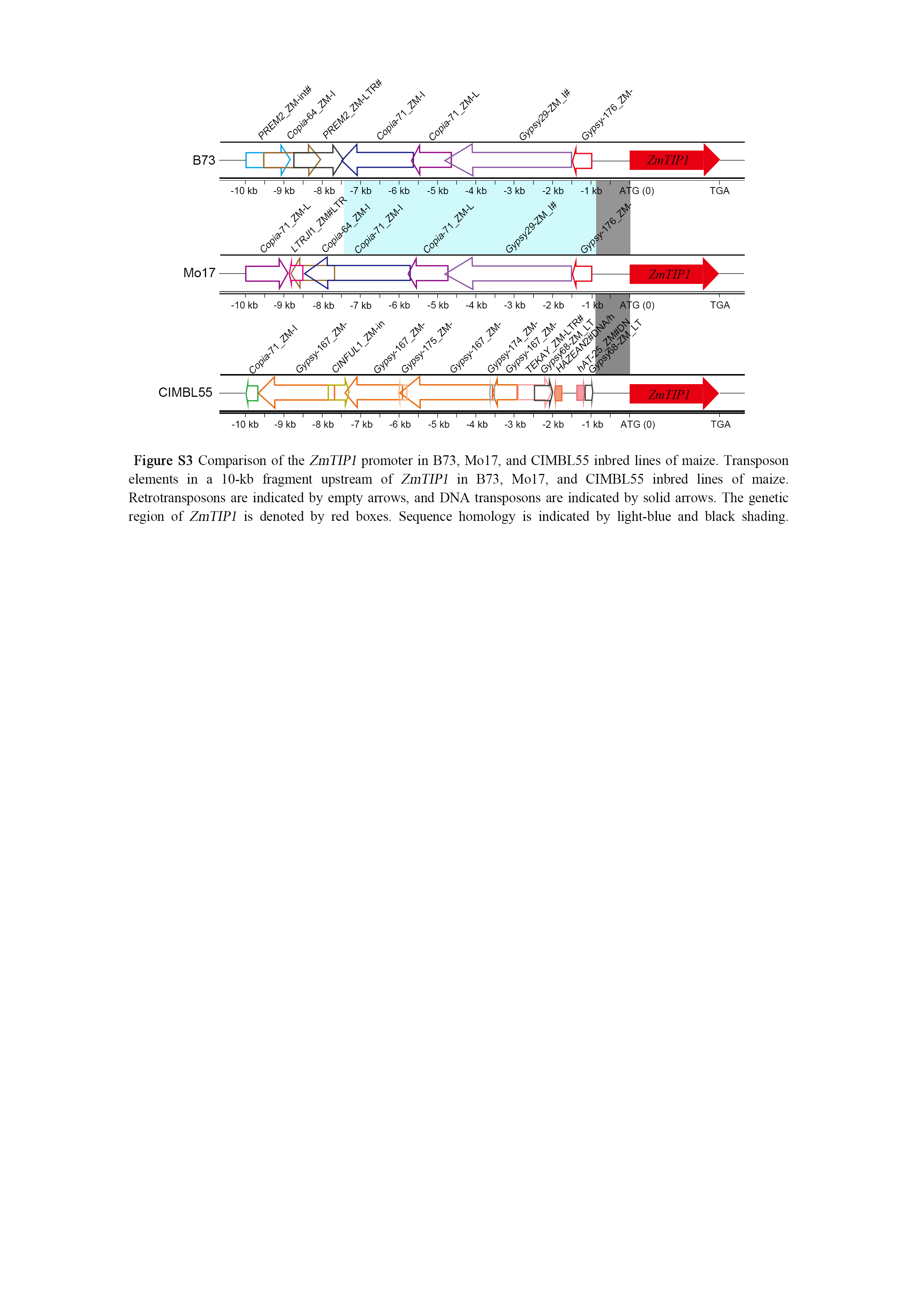

Supplement: Supplementary file 3 — Figure S3 Comparison of the ZmTIP1 promoter in B73, Mo17, and CIMBL55 inbred lines of maize. [file PBI-18-1271-s004.tif]

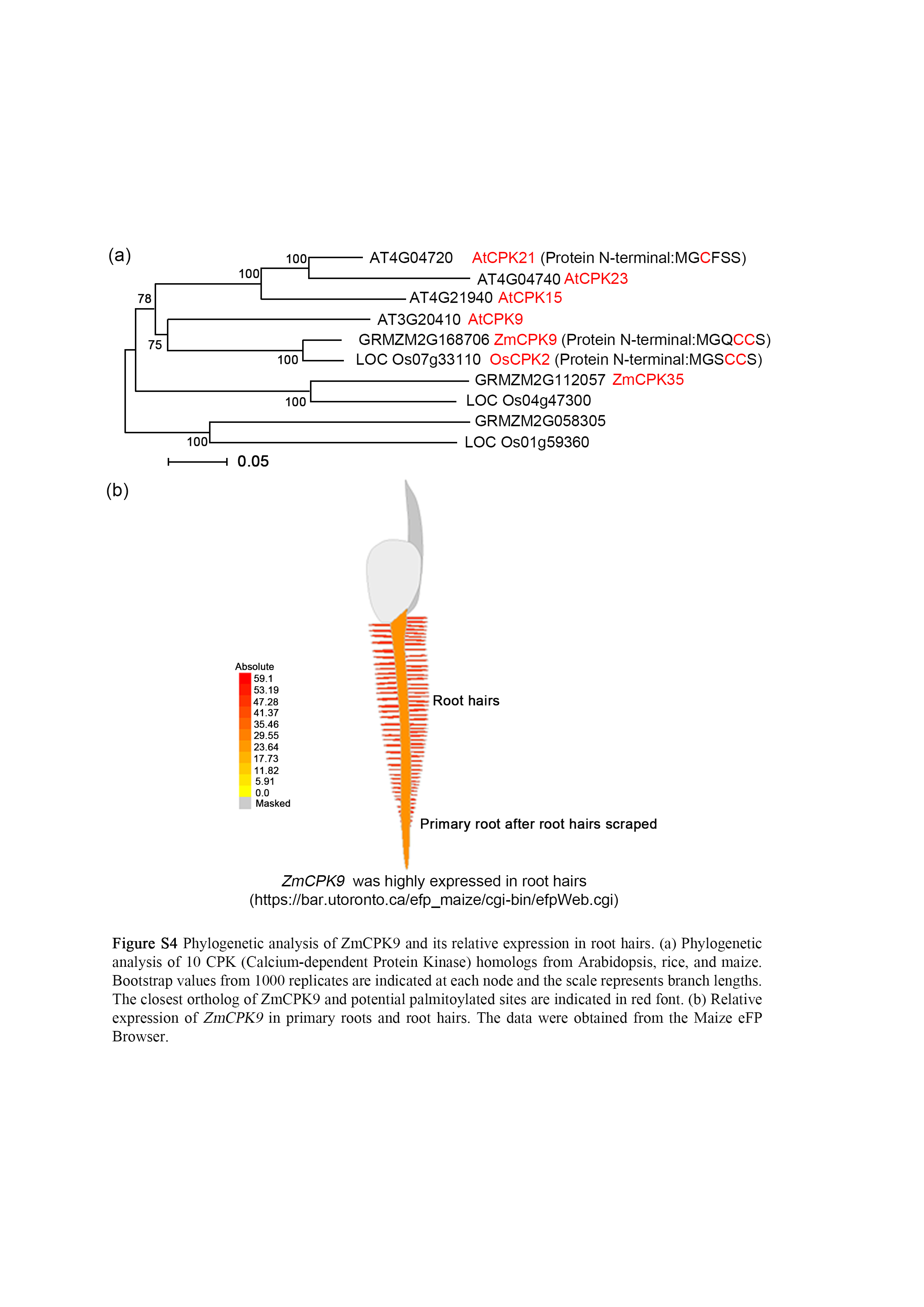

Supplement: Supplementary file 4 — Figure S4 Phylogenetic analysis of ZmCPK9 and its relative expression in root hairs. [file PBI-18-1271-s007.tif]
